# Assessing value‐based health care delivery for haemodialysis

**DOI:** 10.1111/jep.12483

**Published:** 2015-12-11

**Authors:** Eduardo Parra, María Dolores Arenas, Manuel Alonso, María Fernanda Martínez, Ángel Gamen, Juan Aguarón, María Teresa Escobar, José María Moreno‐Jiménez, Fernando Alvarez‐Ude

**Affiliations:** ^1^ Hospital Reina Sofía Tudela Tudela Navarra Spain; ^2^ Hospital Perpetuo Socorro Alicante Spain; ^3^ Hospital Valle del Nalón Langreo Asturias Spain; ^4^ Hospital Casa de Salud Valencia Spain; ^5^ Zaragoza University Zaragoza Spain; ^6^ Hospital General Segovia Spain

**Keywords:** delivery of health care, health care quality assessment, outcome assessment, renal dialysis, social values

## Abstract

**Rationale, aims and objectives:**

Disparities in haemodialysis outcomes among centres have been well‐documented. Besides, attempts to assess haemodialysis results have been based on non‐comprehensive methodologies. This study aimed to develop a comprehensive methodology for assessing haemodialysis centres, based on the value of health care. The value of health care is defined as the patient benefit from a specific medical intervention per monetary unit invested (Value = Patient Benefit/Cost). This study assessed the value of health care and ranked different haemodialysis centres.

**Method:**

A nephrology quality management group identified the criteria for the assessment. An expert group composed of stakeholders (patients, clinicians and managers) agreed on the weighting of each variable, considering values and preferences. Multi‐criteria methodology was used to analyse the data. Four criteria and their weights were identified: evidence‐based clinical performance measures = 43 points; yearly mortality = 27 points; patient satisfaction = 13 points; and health‐related quality of life = 17 points (100‐point scale). Evidence‐based clinical performance measures included five sub‐criteria, with respective weights, including: dialysis adequacy; haemoglobin concentration; mineral and bone disorders; type of vascular access; and hospitalization rate. The patient benefit was determined from co‐morbidity–adjusted results and corresponding weights. The cost of each centre was calculated as the average amount expended per patient per year.

**Results:**

The study was conducted in five centres (1–5). After adjusting for co‐morbidity, value of health care was calculated, and the centres were ranked. A multi‐way sensitivity analysis that considered different weights (10–60% changes) and costs (changes of 10% in direct and 30% in allocated costs) showed that the methodology was robust. The rankings: 4‐5‐3‐2‐1 and 4‐3‐5‐2‐1 were observed in 62.21% and 21.55%, respectively, of simulations, when weights were varied by 60%.

**Conclusions:**

Value assessments may integrate divergent stakeholder perceptions, create a context for improvement and aid in policy‐making decisions.

## Introduction

Since the emergence of dialysis as a new therapy for chronic renal failure in the mid‐1960s, the need for haemodialysis has presented a challenge for health services around the world. Europe alone has more than 180 000 patients on haemodialysis in 4000 centres [1], and haemodialysis costs range between €30 000 and 47 000 per patient per year [2, 3, 4, 5]. The variability in achieving therapeutic goals across centres has been associated with the heterogeneous quality of care [6]. Although there is concern about outcomes and costs, most attempts to assess haemodialysis results are based on non‐comprehensive methodologies (which do not simultaneously consider a wide‐ranging set of meaningful results, such as quality of life, patient satisfaction and costs), or biased methodologies (which do not include the perspective of stakeholders, such as patients or managers) [7, 8, 9].

Evidence‐based medicine has limitations. Approaches to improving patient care have been identified, but the systems for measuring outcome vary markedly among studies. Moreover, the most common quality measures for haemodialysis, such as haemoglobin (Hb) levels and dialysis dose, may explain only 15% of the variability in morbidity and mortality [10]. This limitation suggests that a new approach, which considers outcomes from a broader perspective, is needed to evaluate health care delivery.

The value of health care is a concept that can maximize favourable outcomes for stakeholders and society and integrate their frequently conflicting interests. Here, ‘value’ is defined as the benefit achieved when patients receive a specific medical intervention per monetary unit invested [11]. The benefit of the intervention should be based on outcome, rather than on process; process is largely related to tactics and is not relevant to patients. The actual value of health care delivered by each dialysis facility is connected to a comprehensive set of outcomes and the cost of operation. There is a need for a methodology that can integrate the perspectives of all stakeholders (principally patients, but also clinicians and managers) to establish relevant outcomes that would provide a more accurate, comprehensive assessment of the treatment [12]. On approach to integrating these perspectives is to use Multi‐criteria Decision Analysis (MCDA) methodologies, such as the Weight Sum Model (WSM). This approach can capture stakeholder preferences and inform health care decisions, based on a value matrix framework [13]. To our knowledge, no previous study has assessed value of health care in treatments for end‐stage renal disease.

The Quality Management Group of the Spanish Society of Nephrology designed the present study to reduce disparities and to contribute to achieving the best possible outcomes for patients and the community. The study objective was to develop a methodology for assessing the value of health care delivered by haemodialysis centres according to a comprehensive, feasible set of outcomes.

## Methods

**Figure 1:**
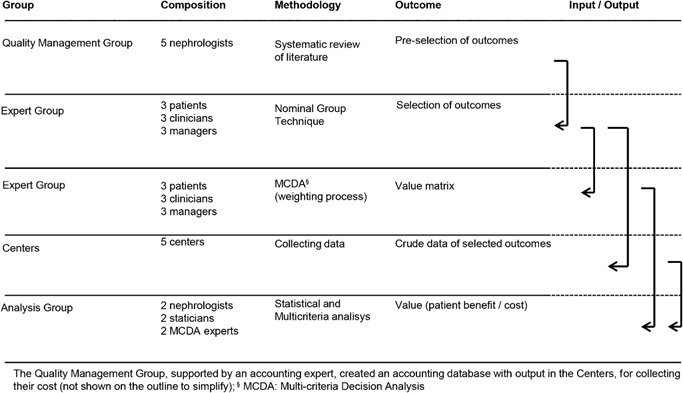
Groups involved in the study. The group composition, methodology, outcomes and sequence of activities are shown with their input and output relationships.

This study was designed to develop a value of health care assessment of haemodialysis. In developing the methodology, different groups contributed, including the Quality Management Group (QM‐G), an Expert Group (EG), the Centres that collected the data and the Analysis Group. Figure 1 shows concisely the action sequence of each group, their composition, methodology, outcomes and relationships.

### Quality management group

In October 2007, a QM‐G meeting was held to develop the pre‐selected outcomes. The QM‐G, composed of five nephrologists, searched the literature to identify indicators commonly used for assessing haemodialysis centres and to pre‐select a set of outcomes. Two reviewers independently searched the literature (Medline, EMBASE and clinical guidelines reports), selected the relevant papers and shared the full‐text articles with the entire QM‐G, which then determined the pre‐selected outcomes by consensus. The criteria for determining the pre‐selected outcomes were that they had to address the stakeholder perspective, be comprehensive and be based mainly on outcomes, rather than on process. The QM‐G decided to include a set of clinical performance measures in the pre‐selected outcomes; however, the consensus required that assessments of clinical results should be based on how closely they were related to morbidity, mortality and the availability of modifiable therapeutic resources. The pre‐selected evidence‐based clinical performance measures fit essentially into clinical guideline recommendations that are graded as level 1, and supported by the quality of evidence as A and B.

According to the concept of value, the haemodialysis cost was considered for every centre. In 2007, the QM‐G, supported by an accounting expert, created an accounting database, specifically designed for the purpose of the study, in Excel® (Microsoft Corporation, Redmond, WA) to record micro‐costing of haemodialysis treatment.

### Expert group

The EG was composed of nine individuals, three patients, three clinicians and three managers who represented the haemodialysis stakeholders. The requirements for the different constituents of the EG were: patients must have been on haemodialysis for at least 3 years and must have served as coordinators for renal patient organizations; the clinicians must have been of recognized prestige and has ample experience – one of these is a nephrologist, one a haemodialysis nurse and one an internal medicine specialist; the clinical managers were selected for three different specialties – economic, medical and health services researcher. A regulated methodology was defined for ensuring the appropriate EG composition [14].

The first task of the EG was to define a comprehensive set of outcomes from haemodialysis treatment, based on the previous pre‐selected outcomes. This set of outcomes was selected with a Nominal Group Technique (November 2007).

The EG members identified an outstanding set of haemodialysis outcomes, or criteria, according to their perceptions and preferences. The group agreed to assess the results by considering four categories: evidence‐based clinical performance measures; mortality; health‐related quality of life (HRQoL), as a patient‐reported outcome measure; and patient satisfaction with the facility, as a patient‐reported experience measure.

### Evidence‐based clinical performance measures

The most relevant clinical measures included dialysis adequacy (Kt/v) [15], Hb concentration [16], serum calcium (Ca), serum phosphorous (P) [17], type of vascular access [18] and the hospitalization rate [19]. The hospitalization rate was selected as a surrogate marker for assessing morbidity. These measures were considered sub‐criteria for assessing evidence‐based clinical performance.

### Mortality

In haemodialysis, mortality is a key outcome in its own right; it reflects both measurable and immeasurable quality aspects. To avoid random variation due to low incidence of an outcome, we evaluated the 2‐year mortality.

### Health‐related quality of life

We decided to measure the HRQoL with the Medical Outcomes Study Short Form 36‐item health survey (SF‐36). This questionnaire captures eight dimensions of HRQoL, grouped into two component summaries: the Physical Component Summary and the Mental Component Summary. The SF‐36 is valid and sensitive [20]; it was also validated in Spanish, and it is the most widely used instrument for evaluating HRQoL in haemodialysis treatment. The SF‐36 results were associated with hospitalization and mortality in patients that received dialysis; the rating scale ranged from 0 to 100 points.

### Patient satisfaction

We implemented a survey that had been transculturally adapted by the QM‐G, based on the Quality of Care in Dialysis Center Questionnaire [21]. The Spanish version of the questionnaire has shown appropriate internal consistency for the quality of care assessment (Cronbach's alpha 0.86). The questionnaire analysed four dimensions: doctor (seven items), nurse (eight items), treatment (eight items) and facilities (eight items). The last page of the questionnaire included a visual analog scale, ranging from 0 to 100, which we used for the purpose of the study.

### Weighting process

The second task of the EG was to perform the process of weighting the criteria and sub‐criteria with the MCDA methodology (December 2010) [22]. The members of the EG settled on the weighting of each variable or criteria of each outcome to build a MCDA value matrix. We previously described the group methodologies for the MCDA and the WSM [14].

In the initial weighting process, each group member distributed 100 points among the four criteria; this was followed by a debate period to discuss differences in opinions. The EG conducted a total of three rounds of weighting, separated by two debate periods. This process allowed the construction of the value matrix framework, according to the WSM. As a result, the means of the final weighted outcomes were: evidence‐based clinical performance measures = 43; yearly mortality = 27; patient satisfaction = 13; and HRQoL = 17. The statistical results showed high intra‐agent agreement (agents were patients, clinicians and managers) and high within‐EG agreement (the EG as a whole was evaluated with a Chi‐squared Pearson test; *P* = 0.841; contingency coefficient, *P* = 0.841). The details of the EG procedure and results were previously published [14]. The values determined in the present study were normalized proportionally to a range of 0 to 100 for clarity.

Subsequently, the EG distributed 43 points in the category of evidence‐based clinical performance measures among the sub‐criteria based on a simplified WSM: each sub‐criterion was assigned a single or double weight. The EG performed this task in a single round to complete the MCDA value matrix. The points were distributed in equal parts (single weights), with the exception of vascular access, which required double weighting. Therefore, the normalized, rounded weightings at the end of the process were: dialysis adequacy = 7 points; haemoglobin concentration = 7; mineral and bone disorder = 7 (serum Ca = 3.5; serum *P* = 3.5); hospitalization rate = 7; and type of vascular access = 15.

### Centres

#### Centre enrollment

During 2007, the QM‐G sent an email to all centres that typically collaborated with the group to invite them formally to participate in the study. All centres that voluntarily accepted were included in this study. We determined that the number of centres that agreed to participate voluntarily was sufficient to achieve the aim of the study. In November 2007, , the EG provided the selected outcome variables to every centre with the directive that each centre would collect the appropriate data starting in 2008.

#### Data collection

In November 2008, we determined the eligibility of cross‐sectional data for the evidence‐based clinical performance measures, HRQoL, patient satisfaction and morbidity. To be included in the analyses, the questionnaires (HRQoL and patient satisfaction) from each centre had to meet a patient response rate of 80%, and missing values in the clinical measures and morbidity could not exceed 5% for each centre. Prospective data were collected for mortality from December 2008 to November 2010.

#### Cost measurement

The accounting department of each centre prospectively collected the data during the financial year of 2008. All centres included the same items and allocation criteria; therefore, costs were comparable among centres. Direct costs, based on invoices, included: staff, consumables, inpatient and outpatient pharmacy, and food. Allocated costs, based on assignments, included: laboratory, diagnostic imaging, patient transport, management, health care equipment and maintenance, and cleaning. The numbers of patients and haemodialysis sessions carried out throughout 2008 were prospectively recorded. This micro‐costing approach allowed each centre to average the total cost, defined as the € expended per patient per year. Hospital admission and vascular access costs were not used to assess value, because these costs were estimated based on the inter‐centre average costs; therefore, these costs were not appropriate for identifying differences between centres. The procedure and results of the cost analysis were published previously [23].

#### Patient morbidity

A modified Charlson co‐morbidity index was collected for each patient. This index was used to adjust for differences in morbidity among centres.

### Analysis group

The analysis group was composed of two nephrologists, two statisticians and two multi‐criteria analysts. The aim of the analysis group was to perform statistical and multi‐criteria analyses to determine the patient benefit and the value assessment.

### Patient benefit

The results from each haemodialysis centre were aggregated into a weighted sum, which was defined as the patient benefit. To obtain the patient benefit in each centre, all criteria were considered. The score for each criterion was proportional to the centre's adjusted co‐morbidity results. First, the overall centre score, or patient benefit, was calculated as the sum of the individual scores for each criteria and sub‐criteria, according to the WSM. Then, the patient benefit results were normalized. Thus, hypothetically, in a centre where 100% of patients underwent autologous vascular access (criterion score = 15), the score assigned to that sub‐criterion would be 15 (15 × 1.00) for the centre. For a continuous variable, such as cumulative survival, we used the average 2‐year survival recorded for each centre; thus, hypothetically, in a centre where 90% of patients survived (criterion score = 27), the score assigned to that sub‐criterion would be 24.3 (27 × 0.90) for the centre.

### Value

Finally, the value of health care for each centre was calculated as follows: Value of Health Care = Patient Benefit/Cost. The value delivered by each centre was obtained by dividing the patient benefit by the average haemodialysis cost (€) at each centre, multiplied by 10 000 (€ per patient per year × 10 000).

According to standard practice for quality measure reporting, we adjusted the value of health care for the case mix, based on demographics and co‐morbidity data. We also conducted a multi‐way sensitivity analysis to explore the value of health care results by considering the key parameters under the most favourable and the most unfavourable conditions.

### Statistical analysis

Patient demographic and clinical characteristics for each centre were summarized with descriptive statistics. Crude comparisons among centres were conducted with the X^2^ test or the Fisher test for categorical variables. We also used the analysis of variance test or the Kruskal–Wallis test, depending on the fulfillment of the required statistical assumptions. The same procedures were used to compare outcomes between groups.

The centre indicators were adjusted for demographic and clinical factors, such as age, sex and Charlson index. Generalized linear mixed models were constructed with these variables as fixed effects and the centre as a random effect. For dichotomous responses, we used the binomial distribution, and for continuous responses, we used the normal distribution. The effect of the centre was checked for significance with the likelihood ratio test, assuming that it comprised a mixture of X^2^ distributions. For comparing cumulative survival after adjustments, first, we used Cox regression models to perform cumulative survival analyses, and then, these models were extended by considering the centre a random effect. Rates were estimated based on all fixed and random effect estimates, and for cases where the random effect variance was estimated to be zero, the same rate was assigned for all centres.

The adjusted indicators (rates and means) derived from the mixed models ranged from 0 to 100, where 100 was the most favourable value. The hospitalization rate was converted to a non‐hospitalization rate to provide positive values. Therefore, to construct the patient benefit of the centre, we summed the indicators for each item, adjusted by its weight, as assigned by the EG.

The statistical package R 2.13.1 was used for statistical analyses. Specifically, we used libraries lme4, survival and Coxme to fit the mixed models.

To account for the uncertainty in weights and costs, we performed a simple sensitivity analysis. In this analysis, the weights and cost of each criterion were randomly perturbed at a specified time by a pre‐established percentage, and the impact of variation on the rank order was assessed. We considered 10 000 simulations, where the criteria and sub‐criteria weights were randomly changed to represent 10–60% of variability; simultaneously, we randomly changed the direct costs by 10%, and the allocated costs by 30%. The analysis was performed with software developed specifically for this simulation, based on the Delphi XE5 integrated development environment.

## Results

Six centres volunteered to participate in the study. One was not included in the analysis because only 47% of patients answered the HRQoL questionnaire; thus, it did not meet the minimum requirement of 80% response rate. The remaining five centres met all requirements and were included in the analysis. Centres 1, 2, 4 and 5 were rural, and centre 3 was urban. Centres 1–3 were integrated into regional hospitals, and centres 4 and 5 were satellite units. Centres 1 and 2 were public and provided direct haemodialysis services (not for profit), and centres 3–5 were state‐subsidized (for profit).

A total of 220 patients were included in the study. Patient characteristics for each centre are given in Table 1. The centres were not significantly different in demographic or clinical features, including co‐morbidity (time on haemodialysis and Charlson index).

**Table 1:** Demographic and clinical characteristics of patients in each centre (C1‐C5)

	C1	C2	C3	C4	C5	*P*‐Value
*n* (%)	41 (18.6)	37 (16.8)	47 (21.4)	54 (24.5)	41 (18.6)	
Gender (men), *n* (%)	25 (61.0)	22 (59.5)	31 (66.0)	34 (63.0)	27 (65.9)	0.964^b^
Age, mean (SD)	67.7 (13.9)	68.4 (13.0)	67.5 (14.7)	64.3 (14.6)	67.8 (15.3)	0.643^c^
Months on HD^a^, mean (SD)	47.6 (41.2)	43.7 (40.4)	43.2 (41.5)	50.0 (52.3)	57.3 (59.5)	0.837^d^
Charlson Index, mean (SD)	7.78 (3.25)	7.68 (2.40)	7.11 (2.06)	7.55 (3.09)	7.21 (3.02)	0.864^d^
**Renal diseases, *n* (%)**						
Unknown	3 (7.3)	4 (10.8)	13 (27.7)	14 (25.9)	6 (14.6)	
Glomerular	9 (22)	11 (29.7)	2 (4.3)	4 (7.4)	5 (12.2)	
Interstitial	5 (12.2)	4 (10.8)	2 (4.3)	5 (9.3)	4 (9.8)	
Polycystic kidney	6 (14.6)	1 (2.7)	4 (8.5)	7 (13)	4 (9.8)	
Vascular‐hypertensive	5 (12.2)	9 (24.3)	11 (23.4)	8 (14.8)	11 (26.8)	
Diabetes nephropathy	5 (12.2)	5 (13.5)	12 (25.5)	11 (20.4)	5 (12.2)	
Others	8 (19.5)	3 (8.1)	3 (6.4)	5 (9.3)	6 (14.6)	

a HD: Haemodialysis.

b X^2^ test.

c Analysis of variance test.

d Kruskal–Wallis test.

Renal disease was not analysed for significant differences between groups, due to the small number of patients in each category.

Table 2 shows the percentage of patients in each centre that achieved the evidence‐based clinical performance criteria. The Kt/v values were above 1.4 in the majority of the patients (>70%) in all centres, except Centre 3. The percentage of patients with functioning autologous arteriovenous fistula (AAVF) was above 70% in all centres, except in Centre 1. Similar rates and means were found after adjusting for patient demographics and co‐morbidity (Table 3). Significant differences were observed among centres. For example, dialysis adequacy (Kt/v) was achieved about 20% less frequently in Centre 3 than in the other centres (*P* = 0.003). Also, Centre 2 achieved significantly better patient satisfaction than the other centres (*P* = 0.043). The patient benefit was evaluated with adjusted statistics, based on the weights assigned by the EG (Table 3). In a 100‐point scale, scores ranged from 65.97 points (Centre 3, which had the lowest scores for dialysis adequacy and serum phosphorous) to 72.59 points (Centre 4, which had relatively low hospitalization and mortality rates, the best serum phosphorous levels, and the highest percentage of patients with functioning AAVF).

**Table 2:** Outcomes achieved for each centre and statistical comparisons

	Centre					Comparison *P*‐value
	C1	C2	C3	C4	C5	
**Evidence‐based clinical performance measures,** *n* (%)						
Kt/v ≥ 1.4	28 (70.0)	29 (85.3)	22 (51.2)	46 (85.2)	36 (87.8)	<0.001^b^
Hb 11‐13 g/dl^a^	21 (51.2)	23 (62.2)	23 (51.1)	33 (61.1)	23 (56.1)	0.745^b^
Ca 8.4–10 mg/dl	35 (85.4)	32 (86.5)	37 (82.2)	45 (83.3)	29 (70.7)	0.363^b^
P 2.5‐4.5 mg/dl	19 (46.3)	17 (45.9)	18 (40.0)	32 (59.3)	17 (41.5)	0.324^b^
Functioning AAVF	19 (46.3)	27 (73.0)	33 (71.7)	42 (77.8)	31 (75.6)	0.011^b^
1‐year hospitalization rate	20 (48.8)	20 (54.1)	19 (40.4)	16 (29.6)	11 (26.8)	0.045^b^
**Mortality;** % (SD)						
2‐year cumulative survival	67.7 (7.4)	58.4 (8.2)	62.1 (7.3)	78.5 (6.1)	74.1 (7.1)	0.264^d^
**Health related quality of life (HRQoL);** Mean (SD)						
MCS from SF‐36	53.1 (14.6)	46.8 (12.5)	46.3 (15.8)	49.1 (15.1)	51.5 (15.4)	0.393^c^
PCS from SF‐36	31.7 (9.5)	33.0 (7.4)	32.7 (10.3)	35.4 (10.0)	36.0 (9.2)	0.340^c^
**Patient satisfaction;** Mean (SD)						
DCQ	91.6 (10.3)	97.6 (6.0)	88.4 (18.5)	87.2 (16.4)	86.8 (12.5)	0.018^c^

a According to the recommendations at the moment data were collected.

b X^2^ test.

c Analysis of variance test.

d Kaplan–Meier log‐rank test.

AAVF, Autologous arteriovenous fistula; Ca, Serum calcium; DCQ, Quality of Care in Dialysis Centre Questionnaire; Hb, Haemoglobin concentration; Kt/v, Dialysis adequacy was calculated with the single pool Daugirdas II method; MCS, Mental component summary from SF‐36 Questionnaire; P, Serum phosphorous; PCS, Physical component summary from SF‐36 Questionnaire.

**Table 3:** Outcomes for each centre, adjusted for demographics (age and gender) and co‐morbidity features (months on haemodialysis and Charlson index)

Outcomes	Weight	Adjusted rates					Comparison	
	(%)	**C1**	**C2**	**C3**	**C4**	**C5**	LR^d^	*P*‐value
**Evidence‐based clinical performance criteria**	**43**							
Kt/v ≥ 1.4	7	72.91	84.16	56.73^a^	85.61	86.70	10.79	0.003
Hb 11‐13 g/dl^c^	7	56.80	56.80	56.80	56.80	56.80	0.00	1.000
Ca 8.4–10 mg/dl^c^	3.5	81.70	81.70	81.70	81.70	81.70	0.00	1.000
P 2.5‐4.5 mg/dl	3.5	46.96	46.83	45.47	51.12	45.86	0.23	0.762
Functioning AAVF	15	57.07	73.16	71.18	75.73	73.62	2.66	0.184
Non‐hospitalization rate (1 year)^b^	7	56.82	54.56	59.63	65.90	65.93	1.09	0.438
**Mortality**	**27**							
Cumulative survival (2 years)	27	78.40	70.60	74.20	86.80	83.70	0.58	0.596
**Health related quality of life (HRQoL)**	**17**							
MCS from SF‐36 Mean^c^	8.5	49.65	49.65	49.65	49.65	49.65	2.82	0.169
PCS from SF‐36 Mean^c^	8.5	33.85	33.85	33.85	33.85	33.85	0.00	1.000
**Patient satisfaction**	**13**							
DCQ Mean	13	91.24	94.54^a^	89.15	88.22	87.85	5.41	0.043
**Patient Benefit (PB)**		**66.25**	**67.61**	**65.97**	**72.59**	**71.28**		
**Cost (€ per patient per year)**		**42 574**	**39 289**	**32 872**	**35 461**	**35 294**		
Direct cost		34 247	31 044	22 174	26 497	26 350		
Allocated cost		8 327	8 246	10 698	8 964	8 945		
**Value (PB/cost) × 10 000**		**15.56**	**17.21**	**20.07**	**20.47**	**20.20**		

a *P* < 0.05.

b Values were converted to positive values (100‐value), which reflect the non‐hospitalization rates.

c Estimate for the random effect with variance equal to zero.

d LR: Likelihood ratio test for models with and without random effects.

AAVF, Autologous arteriovenous fistula; Ca, Serum calcium; DCQ, Quality of Care in Dialysis Centre Questionnaire; Hb, Haemoglobin concentration; Kt/v, Dialysis adequacy calculated with the single pool Daugirdas II method; MCS, Mental component summary from SF‐36 Questionnaire; P, Serum phosphorous; PCS, Physical component summary from SF‐36 Questionnaire.

The value delivered among these five centres ranged from 15.56 (Centre 1, which reflected the highest cost), to 20.47 (which reflected the best patient benefit and intermediate cost).

Table 4 summarizes the sensitivity analysis. The centre rankings of 4‐5‐3‐2‐1 and 4‐3‐5‐2‐1 were very stable; they remained consistent in approximately 63 and 24% of the simulations, respectively. Combined, those rankings appeared in 89.88% of simulations when the weights were varied by 30%. All simulations indicated that centres 2 and 1 delivered the lowest value.

**Table 4:** Multi‐way sensitivity analysis of estimated centre values; the weights and costs (direct and allocated) were changed simultaneously to evaluate the frequency of centre ranking and the best and worst values estimated for each centre

Change	**Centre ranking** (frequency %)			**Centre**					
%weight*	4‐5‐3‐2‐1	4‐3‐5‐2‐1	3‐4‐5‐2‐1	**Scenario**	**C1**	**C2**	**C3**	**C4**	**C5**
10 [10; 30]	64.40	27.04	8.56	Best	18.18	20.16	24.09	24.17	23.85
				Worst	13.55	14.98	17.12	17.63	17.40
20 [10; 30]	64.63	25.90	9.47	Best	18.20	20.22	24.02	24.24	23.90
				Worst	13.45	14.82	16.96	17.45	17.22
30 [10; 30]	63.99	25.89	10.12	Best	18.69	20.54	24.77	24.83	24.45
				Worst	13.28	14.66	16.80	17.12	16.91
40 [10; 30]	63.75	24.15	12.10	Best	18.98	20.75	24.73	25.12	24.77
				Worst	13.14	14.47	16.41	17.12	16.93
50 [10; 30]	62.95	22.67	14.38	Best	18.89	20.88	24.81	25.04	24.66
				Worst	12.87	14.44	16.43	16.82	16.61
60 [10; 30]	62.21	21.55	16.24	Best	18.96	21.12	24.92	25.23	24.89
				Worst	12.35	13.99	16.12	16.28	16.07

*The weights were changed from 10–60%; in all cases, the direct cost was changed by 10% and the allocated cost was changed by 30%.

## Discussion

Our study supported the notion that haemodialysis centres could be assessed according to the value generated for patients and society. The value arises from a meaningful set of outcomes, based on a valid, reliable methodology that reflects the preferences of the principal agents involved in the therapy. The value of the health care delivered by haemodialysis centres should be relevant to stakeholders. This evaluation may lead to substantial opportunities; for example, it may create a context for treatment improvements, process innovations, oversight administration, pay for performance, public reporting and transparency, and health care improvements. The value assessment could also support policy makers in allocating scarce health care resources to maximize care and promote a healthy society.

The results of our study were easily interpreted. Centre 4 delivered the highest value, because it achieved better outcomes for each monetary unit invested. The overall result was that the highest patient benefit (72.59) that could be achieved at the lowest cost provided the greatest value. Although small number of centres included in this study did not allow generalization of the results, it was notable that the three centres that provided the most value were state subsidized, and not the public centres, which provided direct service. The results can be easily understood by patients, haemodialysis staff, managers and policy makers. Thus, to increase their value, haemodialysis centres must improve the patient benefit, reduce costs, or both.

Hypothetically, a properly selected haemodialysis indicator and reliable measures of stakeholder values and priorities would allow construction of a mathematical formula that could be included in the value matrix, and the centre results could be analysed according to that measure. The interpretation would be straightforward, transparent, comprehensive, consistent, reproducible, acceptable to stakeholders, useful for benchmarking and focused on centre improvement; however, the actual utility of this approach, based on values and preferences, merits careful assessment. The complexity of medicine demands a mixture of multidimensional outcomes to evaluate the results of health interventions, which should integrate evidence‐based and preference‐based approaches.

The value methodology encourages clinicians to produce circumstances that are most relevant to patients. However, the value perceived by the patient does not necessarily always relate to evidence‐based medicine; for example, value also includes patient satisfaction with the centre. Consequently, the methodology requires interdisciplinary participation, and it should integrate different points of view, beliefs and experiences.

The MCDA methodology incorporated several assumptions regarding the criteria. First, it assumed preferential independence; that is, it assumed that the decision could be made by using only the criteria on which the alternatives differ. Second, it assumed a trade‐off condition, where adding weight to one criterion should be compensated by reducing the weight given to other criteria. If two hypothetical centres had equal outcomes, except for the dialysis dose (e.g., 80% vs. 70% adequacy), and the 1‐year survival (e.g., 70% vs. 80%, respectively), the decision ranking could be established based on these differences, because the weight assigned to the dialysis dose was less than the weight assigned to the 1‐year survival (7% vs. 27%); therefore, the second centre would exhibit higher value. The MCDA provided a valid methodology, supported by complex sources full of details and speakers. However, there are different methodologies that can incorporate the stakeholder views, such us the Delphi technique. Nevertheless, we suggest that the MCDA is best‐suited to the purpose, because the MCDA includes a face‐to‐face discussion group, and the interaction among participants may enrich their learning, knowledge and reasoning methods. The MCDA can achieve valuable outcomes, and in generating the patient benefit, it unites stakeholder interests [13, 23, 24].

Porter [11] suggested that measuring costs at the individual level, rather than averaging them, could increase accuracy. However, in a homogeneous process like haemodialysis, that policy may not be necessary; it could increase transaction costs, and it would be difficult to implement in a national health service context. We also would like to emphasize that, to achieve value, the outcomes should reflect mainly those that matter to patients. Therefore, we assumed that considering the weighting of all stakeholders could achieve a more comprehensive assessment. Furthermore, we propose that holding the EG meeting and implementing the MCDA methodology placed the members in an appropriate environment for promoting reflection and deep understanding of the outcome significance.

Most previous studies that analysed outcomes from several institutions aimed to grasp the variability among geographic areas or to consider different organizational arrangements. An example of that type of study was the Dialysis Outcomes and Practice Patterns Study, which collected random samples from units in more than 20 countries [5, 9]. Very few studies focused on a key outcome of specific centres [7, 8]. Alternatively, the End Stage Renal Disease Quality Incentive Program of the U.S.A. analysed several key quality measures, including patient‐reported experience measures, and they linked a portion of the payment awarded to centres based on their outcomes (it was a pay‐for‐performance strategy, or value‐based purchasing program). The UK renal registry constitutes an important advance in this area. It is currently establishing routine assessment of patient‐reported outcome and experience measures (the strategy has shifted from working ‘for’ patients to working ‘with’ patients); however, it continues to ignore the cost, which is a key outcome, according to the concept of value. Moreover, no previous study has attempted to put together a comprehensive patient benefit, which could result in better decision‐making in qualifying centres. Patient benefit can enable patient participation in health care decisions and encourage managers and staff to improve the quality of health care. The patient benefit instrument also allows parsing the patient benefit into different indicators for more accurate evaluations, which could facilitate the implementation of targeted activities to improve centre quality.

To our knowledge, no previous study has used a similar methodology to assess the value of other specific processes involved in health care. To that end, this approach, with appropriate modifications, could be tested in other health care domains.

This study included a small number of centres, which prevented the generalization of results. However, the main objective of the study was to develop an instrument to measure the value delivered; therefore, the overall approach represents a significant advance in value assessment methodology. There is no generally accepted questionnaire for assessing patient satisfaction in haemodialysis centres in the Spanish language. Furthermore, there is no consensus in Europe about which specific instrument should be recommended to measure patient‐reported experience. This lack of standardization constitutes a limitation of this study, and it also limits the extension of this methodology to other areas. A recent expert consensus meeting in Europe has declared that more work was needed to resolve this issue [25]. When the cost measurement is based on averages, it may require some adjustment, when centres are not comparable in the extent of morbidity. This problem may be resolved by either calculating individual costs or calculating a cost coefficient related to morbidity. Also, the EG and the weighting structure we described may not be applicable to other cultural or socio‐economic settings; therefore, we encourage testing these components before implementing the approach elsewhere. We are currently attempting to validate this key point by repeating the MCDA procedure in different intra‐ and inter‐cultural groups, and simultaneously, by corroborating results with two different methodologies (WSM and the Analytic Hierarchy Process). Despite the limitations of this value assessment approach, we consider it a useful framework for future investigations related to value insights in a variety of homogeneous processes. Finally, although this is a rational approach for assessing centres, it requires further testing to determine whether it provides any advantages over other evaluation systems.

Our results supported the notion that value can be assessed in haemodialysis centres based on a comprehensive set of outcomes, which include meaningful clinical results, mortality, HRQoL, patient satisfaction and costs. This evaluation may integrate divergent stakeholder perceptions and interests, create context and incentives for quality improvements and facilitate policy‐making decisions. Despite the rationality of the instrument, it requires further testing to determine the possible advantages of this value assessment over other evaluation systems.
